# Red cell membrane protein abnormalities as defined by sds-page among patients with anemia in a West African region hospital practice

**DOI:** 10.22088/cjim.11.3.283

**Published:** 2020-05

**Authors:** Adedoyin Dosunmu, Ebele Uche, Bodunrin Osikomaiya, Ayobami Ismail, Akinsegun Akinbami, Alani Akanmu

**Affiliations:** 1 Department of Hematology and Blood Transfusion, Lagos State University College of Medicine, Lagos, Nigeria; 2 Department of Hematology and Blood Transfusion, General Hospital, Gbagada, Lagos, Nigeria

**Keywords:** Red cell membrane proteins, Sodium dodecyl sulfate-Polyacrylamide gel electrophoresis, Spectrin protein defects

## Abstract

**Background:**

Erythrocytes require an ability to deform and withstand shear stress while negotiating microcirculation. These properties are largely due to their excess surface area per volume and the characteristics of the membrane’s protein. Deficiencies of these proteins are associated with chronic hemolysis.

**Methods:**

This was a cross-sectional study aimed at determining the prevalence of red cell membrane protein abnormalities as determined by sodium dodecyl sulphate polyacrilamide gel electrophoresis (SDS-PAGE) among patients with anemia attending the outpatient clinics of the hospital.

**Results:**

A total of 823 participants were recruited into the study with a mean age of 34±14 years. There were 410 (49.8%) participants with hematocrit ≥ 36% and 413 with hematocrit ≤ 35.9% of which 192 participants (23.3%) had abnormal red cell indices. Following SDS-PAGE, 21 (10.9%) of the 192 participants had deficient PAGE tracing. Abnormal spectrin band was observed in 17 (81%) of the 21 participants. The hematocrit was significantly lower while the reticulocyte count and red cell distribution width were higher in participants with red cell membrane abnormalities.

**Conclusion:**

One in ten patients with mild anemia and abnormal red cell indices in clinical practice may be having hereditary red cell membrane protein defect. Presence of raised reticulocyte count, family history of mild anemia, increased red cell distribution width and red cell morphology may be used to screen for membrane deficiency.

The red blood cell adaptation is characterized by deformability, elasticity, mechanical resistance to shear force, permeability, cellular self-repulsion, poor adhesive surface and ability to withstand oxidative stress. Most of these characteristics are attributable to the cholesterol mediated fluidity of the lipid bilayer, the meshwork of skeletal membrane proteins and excess of surface area to volume of the cell (1-3). Furthermore, red cell surface has net negative charges due to excess acetylated derivative of neuraminic acid (sialic acid) which prevents clogging of the cells in the capillaries (4, 5). Adherence to other cellular blood elements and the endothelium is prevented by keeping phosphatidylserine in the interior of the lipid bilayer via activities of flippase, scramblases and flippases (6).The lipid bilayer and the skeletal proteins are prevented from oxidative damage by reduced glutathione which is linked to the pentose phosphate pathway while the glycolytic pathway generates a regulated amount of energy sufficient to drive the transmembrane pumps that maintain the osmolarity of the cell (7).

Disruption of these mechanisms leads to cell rigidity and early engulfment by macrophages with a reduction in red blood cell life span. These underscore the importance of the red blood cell membranes especially the membrane proteins. The hetero-dimer, alpha / beta spectrin, which forms a hexagonal mesh of skeletal protein is linked to a multiple transmembrane protein (band 3) via ankyrin (8, 9, 10). Binding of protein 4-2 strengthens this complex which forms the vertical support for the lipid bilayer that prevents the loss of membrane material by vesiculation and hence spherocytosis (8, 9, 10). The spectrin hetero dimers form head to head tetramers while actin filaments join transmembrane glycophorin C to these junctions which are strengthened and made more flexible by attachment of protein 4.1, adducin, and tropomodulin (11-14).

Lesions affecting this complex cause loss of membrane elasticity and the cell retains an elongated shape after passing through the capillaries to form elliptocytes with reduction in red cell life span (13-17).In severe forms, the red cell fragments easily and presents as hereditary pyropoikilocytosis where elliptocyte, spherocytes and fragmented cells are seen in blood film (18). Defect in band 3 is associated with the Southeast Asia ovalocytosis which is common (up to 35%) in Melanesia (19). Ionic balance defects like: mechanosensory protein (PIEZO1) and the Gardos channel (KCNN4) defects are associated with dehydrated hereditary stomatocytosis 1 and 2, respectively; Rh-associated glycoprotein (RhAG) defects with overhydrated stomatocytosis; anion transporter (SLC4A1) defects with increased permeability to cations at low temperatures; adenosine triphosphate-binding cassette family member (ABCB6) defects with familial pseudohyperkalamia while glucose transporter(GLUT1) defects manifest with mental retardation, seizures, hepatosplenomegaly and cryohydrocytosis (20, 21, 22). Changes in mean cell volume (MCV), mean cell hemoglobin (MCH), mean cell hemoglobin concentration (MCHC) are characteristics of defects in red cell ion regulation (22).

The clinical presentations of hereditary membrane protein defects vary from asymptomatic to chronic anemia with reticulocytosis, jaundice, splenomegaly, gallstone formation and neonatal jaundice while very severe lesions may present with hydrops fetalis or intra uterine fetal death (22). The British committee on hematology guideline of 2011 recommends that patients with these clinical presentations and a family history of hemolytic anemia which is not auto-immune should be screened for red cell membrane protein abnormality using a combination of acidified glycerol lysis time test (AGLT) or flow cytometric osmotic fragility test combined with eosin-5-maleimide binding test (EMA) (23). Other tests are the sodium dodecyl sulphate polyacrilamide gel electrophoresis (SDS-PAGE) or ektacytometry (23). Next Generation Gene Sequencing (NGS) is more specific but is hardly necessary in clinical practice (23).Inherited red cell membrane disorders are more common in malaria endemic zones, where these tests are not readily available (17, 23).Unfortunately, clinicians rarely request for these tests especially in resource poor countries of West African sub region where hereditary elliptocytosis is more common. The use of EMA is not applicable in hereditary elliptocytosis, osmotic fragility tests have low sensitivity and specificity while ektacytometry and flow cytometry are not readily available in resource poor countries. This study, therefore, sought to identify the burden of red cell membrane abnormalities among patients attending the outpatient department of the hospital by using available screening method. SDS-PAGE was chosen to determine specifically red cell membrane protein deficiencies though it may not detect some qualitative changes. This should raise clinical awareness among physicians in our secondary and tertiary hospitals and assist in developing a diagnostic algorithm in this geographical zone.

## Methods

The study was done over a period of 6 months in 2016 at the outpatient clinics of the Lagos State University Teaching Hospital after obtaining ethical approval from the hospital research and ethics committee.

This was a cross sectional multistage study. Participants (823) were subjects that gave written informed consent from the family medicine clinic, family planning clinic, medical out- patient clinic and the blood donor clinic of the hospital. Blood samples of participants (192) that had hematocrit below 36% and reticulocytes (≥2%), abnormal mean cell volume (MCV) <80 fl or >98fl, high mean cell hemoglobin concentration (MCHC) ≥ 36g/l or red cell distribution width with coefficientof variation (RDW) ≥ 15% were further tested for red cell membrane protein defects using sodium dodecyl sulphate polyacrilamide gel electrophoresis. Inclusion criteria were subjects who gave consent to participate in the study and subjects above the age of 16 years irrespective of the sex. Exclusion criteria were subjects with evidence of organ damage e.g. liver failure, renal failure, bone marrow failure from past medical records; those who have been recently transfused (within 6 weeks); subjects on medications that can cause hemolysis like anti-malarias, aldomet, cephalosporins or high dose penicillin and subjects with known history of hemoglobinopathy of regional significance. The minimum sample size of 753 was determined using the formulae for cross-sectional qualitative studies: n= Z_1-_**α**^2^P (1-P)/D^2^ with a prevalence of 2% (24). Trained phlebotomist collected 4 mls of venous blood from the participants into 2 EDTA sample bottles each. Full blood counts were done from one sample bottle using the 5 part hem-autoanalyzer (Mindray BC-5300 made in China) within 2 hours of collection. The blood films were stained with May Grunwald Giemsa stain and read under the microscope by two experienced hematologist simultaneously. The reticulocyte counts were done manually by two experienced medical laboratory scientists simultaneously using new methylene blue stain. 

Corrected reticulocyte count^ 25 ^ = reticulocyte count (%) x Packed Cell Volume of patient in % /45 % (Normal)

Average PCV for Red blood cells for SDS-PAGE were separated from 2 ml of blood collected in EDTA tubes by density gradient centrifugation at 450 x g at room temperature for 30 min. 

**FIGURE 1:**
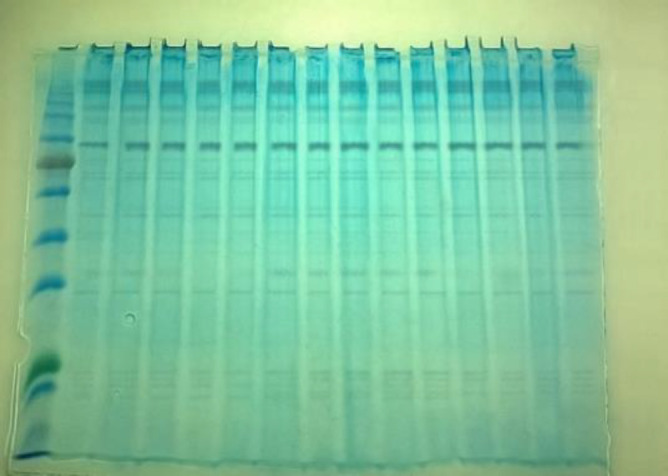
SDS PAGE Gel of control subjects

**FIGURE 2:**
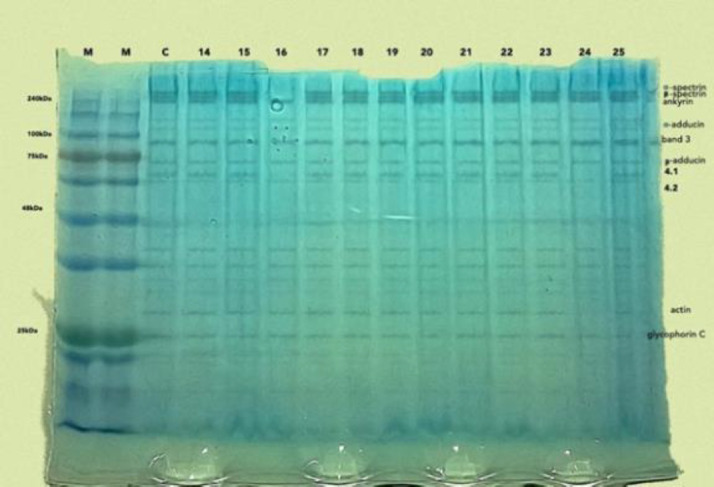
SDS PAGE gel of subjects with PCV<36% and abnormal red indices

The red blood cell pellets were washed using phosphate buffered saline (PBS) by centrifugation for 10 min. Red blood cell ghosts were prepared as described by Hanahan and Ekholm (26).Red blood cell ghosts were pelleted by centrifugation at 20,000xg at 4^o^C for 45 min in an Eppendorf Centrifuge 5430R (Eppendorf AG, Hamburg Germany). Pellets were washed and vortexed for two minutes using cold 20 imosM Tris-HCl then centrifuged at 20,000xg at 4^o^C for 45 min. Red cell membrane ghosts were resuspended in 20 imosM Tris-HCl and 50ul of protease inhibitor cocktail (Carl RothGmbH Co.KG) and stored at -80^o^c until it was ready to be run in the SDS PAGE electrophoresis (27). Samples to be separated by SDS-PAGE were treated as follows: ^28^ 10μl of loading buffer (containing 1μl -mercaptoethanol, 20μl SDS and 20μl bromophenol blue) were added to 30μl of RBC membrane proteins and the mixture was heated for 10 min at 70˚C. The samples were loaded onto a precast, gradient, 12% polyacrylamide gel after optimization using MOPS running buffer containing 4μl of reducing agent (1×), as per manufacturer's instructions. The gel was fixed in a 40% methanol, 10% acetic acid solution and stained with colloidal Coomassie blue to visualize protein migration. The test samples were controlled by adding a protein ladder (Roti®- Mark Tricolour, Carl RothGmbH Co.KG) in the first well and a normal sample from a healthy blood donor with normal red cell indices which has been previously run on SDS PAGE to confirm absence of any defect. Preparation of the ghost cells and electrophoresis were done at the Nigerian Institute of Medical Research. The gels were read, before and after enhancement with photoshop, by two independent investigators. A missing band is considered absent and faint band is considered deficient. All control samples done to optimize the method had visible bands on the gels in the α-spectrin, β-spectrin, ankyrin, α- adducin, band 3,-adducin, protein 4.1, protein 4.2, actin and glycophorin C protein regions (figures 1 and 2).

**Data analysis: **The data were recorded and analyzed with SPSS Version 20 software. The mean, median, standard deviation and other parameters of statistical importance were generated as necessary for continuous data. Tests of statistical significance between variables included chi-square analysis, t-test and Fischer**’**s exact for discrete data. Level of significance was set at p<0.05.

## Results

A total of 823 participants were recruited into the study consisting of 203 participants from the medical out-patient clinics, 230 participants from the family medicine clinics, 190 participants from the family planning/infertility clinics, 200 participants from the Blood donor clinics who failed blood donor fitness test (i.e. hematocrit less than 39% for males and 36% for females). There were 469 (53.3%) males and 411 (46.7%) females. The mean age was 34±14 years. Of the 823 participants that were screened, 410 (49.8%) had their hematocrit greater than 36% and were not further screened for red cell membrane protein abnormalities. Among the 413 subjects with a hematocrit less than 36%, 221(53.5%) subjects had normal reticulocyte count and red cell indices (i.e. reticulocyte count, mean cell volume, mean cell hemoglobin, mean cell hemoglobin concentration and red cell distribution width). 

The rest, 192 (413-221=192; 46.5%), study participants with a hematocrit less than 36% and abnormal red cell indices, constituted the selected group whose samples were further subjected to red cell morphology and sodium dodecyl sulfate-polyacrylamide gel electrophoresis.There were 21 (10.9%) of the selected 192 participants or 2.5% of total participants with abnormal red cell membrane proteins following SDS PAGE. On microscopy, all the 192 participants had either hypochromia, microcytic red cells or polychromasia. The number of participants with a percentage of elliptocyte <25% was 176 (91.7%) and only 12 of them had abnormal membrane proteins. All the participants with elliptocytosis > 50% on blood film (4) had membrane protein abnormalities while 4 out of 11 participants with elliptocytosis between 26% and 49% had abnormal membrane proteins. Only one participant had microspherocytes (table 1). Among the 192 participants with a hematocrit less than 36.0%, the mean hemoglobin concentration (Hb =10.2±1.3g/dl) and hematocrit (32.1±3.8%) were significantly higher in patients without red cell membrane defects than those with red cell membrane defects (table 2).

 The mean corpuscular volume (MCV= 85.2±12.7fl) and mean corpuscular hemoglobin (MCH=26.4±4.4 pg) in participants without red cell membrane defects were higher than in patients with red cell membrane defects. However, this difference did not reach significant levels (table 2). The mean corpuscular hemoglobin concentration (MCHC) of participants with or without red cell membrane defects were essentially the same while the corrected reticulocyte count (2.9±0.6%) and red cell distribution width (RDW=29.0±6.6) were significantly higher in patients with red cell membrane defects than those without red cell membrane defects (table 2). The most common deficiency seen on SDS-PAGE was in the β-spectrin band which was abnormal in 10 of 21 participants (47.6%) and this was followed by deficiency in protein 4.1band (Table 3).

**Table 1:** Red cell morphology in patients with abnormal red cell proteins

**Red cell** **Morphology**	**Red cell** **Membrane protein**		**Total**
	**Abnormal**	**Normal**	
Polychromasia,elliptocytes (<25%)	12 (57.2%)	164 (95.9%)	176 (91.7%)
Polychromasia,elliptocytes (26%-49%)	4 (19.0%)	7 (4.1%)	11 (5.8%)
Polychromasia,elliptocytes (50%-75%)	2 (9.5%)	0 (0.0%)	2 (1.0%)
Polychromasia, Elliptocytes (>76%)	2 (9.5%)	0 (0.0%)	2 (1.0%)
Spherocytosis	0 (0.0%)	1 (0.5%)	1 (4.8%)
Total	21 (100.0%)	171 (100.0%)	192 (100.0%)

**Table 2:** Abnormal red cell membrane proteins and Full Blood Count Parameters

**Red cell parameters** **(Mean±SD)**	**Participans with abnormal red cell membrane** **Abnormal (21) Normal (171)**		**p-value**
PCV^++^ (%)	29.2±4.4	32.1±3.8	<0.05
Hb^+++^ (g/dl)	9.2±1.8	10.2±1.3	<0.05
Corrected reticulocyte count	2.9±0.6	1.6±0.5	<0.05
Red cell distribution width	29.0±6.4	16.8±1.9	<0.05
Mean corpuscular volume(fl)	80.0±14.0	85.2±12.7	0.08
Mean corpuscularHaemoglobin(pg)	24.7±4.7	26.4±4.4	0.09
Mean corpuscularHaemoglobin concentration	30.3±3.7	30.4±2.2	0.87

**Table 3:** Specific Protein Abnormality

**Red Cell Membrane Protein**	**Normal**	**Abnormal**
**α**-SPECTRIN	10110 (47.6%)	11 (52.3%)
-SPECTRIN	9 (42.9%)	12 (57.1%)
ANKYRIN	20 (95.2%)	1(4.8%)
**α**-ADDUCIN	21(100.0%)	0
BAND 3	21(100.0%)	0
-ADDUCIN	21(100.0%)	0
PROTEIN 4.1	11(52.4%)	10 (47.5%)
PROTEIN 4.2	18 (85.7%)	3 (14.3%)
ACTIN	17(81.0%)	4 (19.1%)
GLYCOPHORIN	16 (76.2%)	5 (23.8%)

## Discussion

Approximately, half of the participants had anemia as defined by a hematocrit of 36% (413 vs 823) and a half of them had normochromic normocytic anemia (221 vs 413). The prevalence of anemia was higher in this hospital-based study compared to global finding of 30.2% in non-pregnant women but similar to estimates of 48.5% in Nigeria by IndexMundi of 2006 (28). Among the other half (i.e. 413-221=192), with high reticulocyte count and/or abnormal red cell indices, 10.9% (21) had red cell membrane protein deficiencies as defined by SDS-PAGE. This is 2.5% of the total participants (21 in 823) and is similar to the prevalence of hereditary elliptocytosis (HE) in West Africa (2%) (22). This is also similar to other studies which showed that the prevalence of hereditary red cell membrane protein deficiency in the West African region varies from 0.6% to 3% and that elliptocytosis, an autosomal dominant hereditary disorder, is the common deficiency (29-31).Studies have shown that hereditary spherocytosis is the predominant red cell membrane protein deficiency among Caucasians with a prevalence ranging from 1: 2000 to 1: 5000 (32). The higher prevalence of elliptocytosis in West African sub region has been attributed to malaria which gave the heterozygotes a survival and selective advantage in a malaria zone probably due to loss of re-invasive ability of malaria parasite in cells carrying the heterozygous gene (33). 

Those identified on SDS-PAGE presented with varying degrees of elliptocytes on blood film morphology (table 1). Only 12 participants (6.8%) with <25% elliptocytes on morphology (176) had abnormality on SDS-PAGE yet they represent 57.1% of participants with red cell membrane disorders on SDS-PAGE (table 1: 12 in 21). These suggest that the SDS-PAGE analysis may not identify all cases of elliptocytosis while the contrary may also be valid, that is, the presence of elliptocytes < 25% is not enough to conclude that there is red cell membrane defect (likelihood ratio=0.59). However, all the participants with elliptocytes >50% had membrane protein defects but they were the minority (19% in table 1). This indicates that the presence of >50% elliptocytes on blood film is suggestive of red cell membrane abnormality or polymorphism bearing in mind the report from clinical finding that majority of HE are asymptomatic as only 10% of HE have moderate to severe clinical course (35). 

Moreover, patients with elliptocytosis <25% on blood film should be considered for osmotic fragility test after clinical evaluation.Other parameters with significant differences were corrected reticulocyte count and the red cell distribution widths which were significantly higher in participants with membrane protein disorders (table 2). Therefore, in designing Institutional algorithm for diagnosis of hereditary red cell membrane disorders in the West African region, anemia with reticulocytosis, high red cell distribution width, presence of elliptocytes and positive family history are suggestive of hereditary hemolytic elliptocytosis in the absence of hemoglobinopathy or immune hemolysis. Such cases will require screening tests like osmotic fragility with or without incubation and acidified glycerol lysis test. The most common abnormality on SDS-PAGE in this study was in spectrin band followed by that of protein 4.1 then actin and glycophorin C (table 3). This is similar to the result by Dhermy D *et al (*34*)*. These are proteins involved in horizontal association and therefore cause elliptocytosis. The lack of association of spectrin heterodimers to form tetramers has been linked to elliptocytosis and protein analysis has demonstrated increased dimmers in people of African origin while defect in protein 4.1 is more common among Caucasians (34, 35).

**Limitation of study** **:** Screening tests were limited to red cell morphology, reticulocyte count and red cell indices while osmotic fragility tests with or without incubation was avoided so that readily available tests were utilized. The SDS-PAGE would not detect some qualitative changes in red cell membrane proteins and therefore some anemic patients with membrane disorders could be missed.

In conclusion anemia is a common clinical presentation and about half of these patients will present with abnormal red cell indices. While 10.9% of those with abnormal red cell indices had abnormal SDS-PAGE traces with a predominance of spectrin abnormalities and hereditary elliptocytosis is the most common. Diagnostic algorithm should therefore consider the presence of anemia, reticulocytosis, high red cell distribution width, red cell morphology and family history before proceeding on a combination of osmotic fragility test or/ and acidified glycerol lysis test.
